# Investigating the Effects of Three Needling Parameters (Manipulation, Retention Time, and Insertion Site) on Needling Sensation and Pain Profiles: A Study of Eight Deep Needling Interventions

**DOI:** 10.1155/2013/136763

**Published:** 2013-09-18

**Authors:** Bertrand Y. K. Loyeung, Deirdre M. Cobbin

**Affiliations:** School of Medical and Molecular Biosciences, Faculty of Science, University of Technology, Sydney, 15 Broadway, Ultimo, NSW 2007, Australia

## Abstract

*Introduction*. In traditional Chinese acupuncture, needle sensation (*deqi*) is purported to contribute to a therapeutic outcome. While researchers have attempted to define *deqi* qualitatively, few have examined the effects of needling parameters on its intensity. *Methods*. 24 healthy subjects completed eight interventions scheduled at least one week apart, which involved manual acupuncture to LI4 or a designated nonacupoint (NAP) on the hand, with real or simulated manipulation each three minutes and needle retentions of one or 21 minutes. Intensities of needling sensation and pain were reported every three minutes and sensation qualities were reported post-intervention. *Results*. Immediately after needle insertion, similar levels of mean needle sensation and of pain were reported independent of intervention. At subsequent measurement times, only two interventions (one at LI4 and one at NAP) maintained statistically significantly elevated needle sensation and pain scores and reported higher numbers of needle sensation descriptors. For both, the needle was retained for 21 minutes and manipulated every three minutes. Neither intervention differed significantly in terms of levels of pain, and needle sensation or numbers and qualities of needle sensation described. *Conclusion*. In this group of healthy subjects, the initial needling for all eight interventions elicited similar levels of needle sensation and pain. These levels were only maintained if there was ongoing of needle manipulation and retention of the needle. By contrast, the strength of needle sensation or pain experienced was independent of insertion site.

## 1. Introduction

Many traditional Chinese acupuncturists consider the elicitation of *deqi* (needling sensation) during needling as essential for a therapeutic outcome [1]. *Deqi* is often described by acupuncture recipients as a constellation of sensations including soreness, numbness, distension, aching, or heaviness [2]. However, it is only in recent decades that research has been undertaken to determine the nature of the *deqi* phenomena and develop reliable instruments to measure and quantify the sensations that arise during acupuncture [3].

Interestingly, on one hand, authors strive to differentiate those needle sensations that they regard as aspects of *deqi* from ones that reflect the acute pain associated with needle insertion and retention. Yet, on the other hand, various psychometric instruments developed to measure the qualities and often the intensities of the *deqi* sensations tend to have much in common with both the content and approaches that characterise the reliable and valid McGill Melzack Pain Questionnaire (MMPQ). This is not unexpected, since Melzack [4, 5] and colleagues cast widely for descriptors (sensory, affective, and evaluative) that people used to describe pain and then grouped them into categories of similar sensation, and within each grouping, ordered the terms from minimally discomforting or painful, through to the most intense.

In an early example, Vincent and colleagues [6] in 1989 adapted the MMPQ to create a new scale of 20 sensory descriptors to measure the sensations of acupuncture. Interestingly, in this study, with a sample of 65 volunteers, needling both at acupoints and nonacupoints provoked similar levels of needle sensation on the scale, suggesting that *deqi* was not exclusive to acupoints. This instrument, as with others that grew out of the MMPQ, has been criticised for these origins from a pain questionnaire and consequently of potentially measuring pain in addition to the supposedly nonpainful sensations arising from acupuncture [7]. 

A range of psychometric instruments to measure *deqi *has subsequently been developed. Common modifications have been to select only a subset of the 20 categories included in the MMPQ and to expand single descriptors from the MMPQ into a Visual Analogue Scale (VAS) or similar scale, where the intensity of that one quality can be further refined: for example, ache, tingling, numbness, with each ranging from none to unbearable [8]. In some instruments, the descriptors have been sourced from subjects after receiving acupuncture. Others have included descriptors selected by acupuncturists. For example, MacPherson and Asghar [9] developed a classification of needle sensations associated with *deqi* based on ratings by 20 TCM acupuncture experts. Two clusters of sensations were identified. One was linked with *deqi* (aching, dull, heavy, numb, radiating, spreading, and tingling) while the other related to acute needling pain (burning, hot, hurting, pinching, pricking, sharp, shocking, stinging, and tender). White and colleagues [10], based on their qualitative interviews with patients, developed the 17-item Southampton Needle Sensation Questionnaire (SNSQ). Kong et al. [8] developed the Massachusetts General Hospital Acupuncture Sensation Scale (MASS) which uses 13 Likert scales for 12 sensory descriptors as well as a scale for other sensations, a mood scale and an acupuncture sensation spreading scale. It has been translated into the Chinese language for use in Asia [11]. Benham et al. [1] used a modified single VAS for recording and monitoring *deqi *sensations while Kou et al. used several scales for recording five *deqi* sensation variables [12]. 

While many authors have attempted to define the qualities that make up the *deqi* experience, few studies have evaluated the influence of needling parameters such as depth of needling, presence or absence of needle manipulation, and duration of needle retention on the presence and maintenance of the *deqi* sensation [1, 13]. The present study examined three such needling parameters in relation to the reporting of *deqi* by healthy subjects as measured by a single VAS. In addition, it reported the qualities of the needle sensation experienced and the intensity of pain at the needling site. The three parameters studied were site of needle insertion, needle manipulation, and duration of needle retention. 

This research comprised one component of a comprehensive examination of the effects of different needling parameters on regional pressure pain threshold. All subjects received the same baseline threshold measurements prior to each needling session: this involved them relaxing supine on a treatment table for ten minutes while pressure pain threshold (identified by the subjects as when the pressure sensation first becomes discomforting) was measured with an algometer at sites on the limbs and head. Ethics approval was obtained from the University of Technology, Sydney (UTS) Human Research Ethics Committee prior to commencing the study (UTS HREC 2009-067A). This study closely follows the design and protocols developed in 1999 at UTS and applied to related research into acupuncture and pressure pain threshold in six previous postgraduate research programs [14, 15].

## 2. Aim

The aim is to investigate the effect of three needling parameters on the strength and quality of *deqi *(henceforth referred to as “needle sensation”) reported and the strength of pain at the needling site. The three parameters comprised site of needle insertion, needle manipulation, and duration of needle retention. Outcome measures were quantitative VAS reporting of intensity of pain and of needle sensation. Qualitative descriptors of the needle sensation were also recorded.

## 3. Methods

### 3.1. Study Design

The study formed one arm of a comprehensive examination of the effects of different acupuncture needling parameters on regional pressure pain threshold in healthy subjects. This needling component of the overall research employed a randomised single blind (subject) within subjects with repeated measures design using a standardised protocol. 

### 3.2. Subjects

The 24 study subjects (12 men and 12 women) were volunteers from the broader university staff and student community, recruited via the UTS Faculty noticeboards and/or word of mouth. Study inclusion criteria were healthy adults with no medical history of chronic musculoskeletal disorder, aged between 18 and 45. Exclusion criteria included regular users of analgesic or other drugs that may dampen pain perception, haemophilia, and use of anticoagulant medication that may interfere with blood clotting. Participants were required to abstain from analgesic medication on experimental intervention days. 

### 3.3. Interventions

For each intervention session, a single 0.22 mm × 30 mm sterile stainless steel disposable needle (Viva USA) was inserted at either the acupoint, LI4 or the nonacupoint, NAP and for either one or 21 minutes. Insertion on all occasions was perpendicular (90°) to the skin and to a depth of 15–20 mm, thereby not only puncturing the skin but also underlying structures such as muscle, fat, and fascia. The intervention was applied unilaterally on the right arm. The needling parameters examined, site of insertion, needle manipulation, and needle retention time, are defined below. 

#### 3.3.1. Site of Needle Insertion

LI4: acupoint, located as the highest point of the *adductor pollicis* muscle when the thumb is adducted [16].

**Figure 1:**
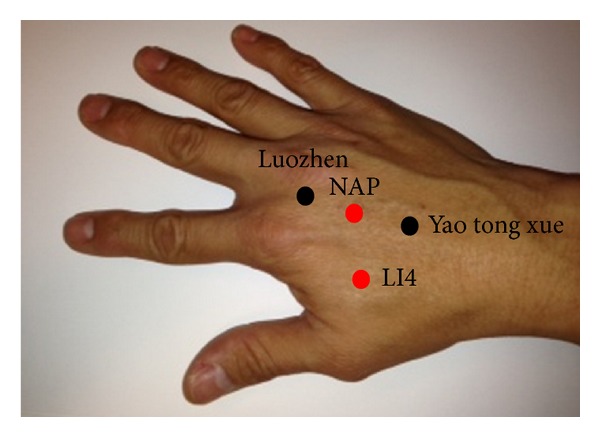
Location of LI4 and NAP in relation to the two extra acupoints *luozhen* and *yao tong xue. *

NAP: nonacupoint located within the same dermatome as LI4, on the dorsal aspect of the hand, midway along the medial shaft of the second metacarpal bone (see Figure 1). This point is equidistant between the two extra acupoints *luozhen* (stiff neck) and *yao tong xue *(low back pain acupoint). No reference to a classical acupoint at this site has been documented [17, 18]. 

#### 3.3.2. Needle Manipulation

Manipulation present—needle manipulation involved rotating the needle for five seconds between the thumb and index finger through a large 540–720° angle in a bidirectional manner. This was applied every three minutes. 

Manipulation absent—every three minutes, the acupuncturist rested his hand in the same position as above and lightly moved his fingers on the back of the subject's hand to mimic movements that would accompany needle manipulation. This is referred to as “simulated manipulation.”

#### 3.3.3. Needle Retention Duration

Duration of needle insertion was either one minute or 21 minutes. Note that for the one minute duration needling interventions, the needle was only present during this initial period (*t* = 0-1). However the acupuncturist applied simulated manipulation of the “virtual” needle every three minutes throughout the 21-minute “intervention” period, as described above. At 21 minutes, the acupuncturist ensured that the “removal” of the “needle” was evident to the subject.

#### 3.3.4. Intervention Parameter Combinations

Each intervention involved deep needle insertion and one of the following eight sets of parameters (Table 1). 

*Outcome Measures*: *Subjects' Perceptions of Intervention Needle Sensation and Pain.* Needling sensation was defined for the subjects as *any sensation other than needling pain*. Subjects quantified the intensity of the needle sensation using a 100 mm VAS designated to range from no sensation/pain to intense sensation/pain. For all interventions, every three minutes, subjects reported in turn: needling sensation (*Do you feel any needling sensation at this point in time*) and pain intensity (*Are you experiencing pain at present*) on a 100 mm VAS with a sliding scale (held up for them by the acupuncturist). These measurements occurred immediately after true or simulated manipulation and the VAS scores were recorded by the acupuncturist. At each measurement occasion, subjects were encouraged to describe the needle sensations they were reporting, as a check on their understanding of reporting needle sensation rather than pain. At the completion of each session, subjects recorded global needle sensation and pain ratings, again using a 100 mm VAS. Where they recorded a needle sensation they included a written description of the sensations experienced.

### 3.4. Intervention Sequence Allocation

Since all subjects received four interventions to each of two needling sites, it was assumed that they would realise that two locations were being used. In previous studies that have used the same protocol and insertion sites, postintervention feedback from subjects (many of whom were final year acupuncture interns at UTS) showed that while most subjects were aware of different locations being needled, both sites were thought to be acupoints and the NAP location was even reported by some subjects to be an extra point [19].

To control for possible changes in expectations that might gradually develop during the eight experimental sessions, careful stratified randomisation of presentation order of interventions was implemented. This included using an 8 × 8 matrix to allocate the order of intervention presentations across the 24 subjects so that there would be equal numbers of subjects completing each intervention at sessions one through to eight. Thus, all interventions were similarly exposed to potential changes in expectations based on the varying extent of exposure by different subjects to the set of interventions.

A random sequencing of the eight interventions for each subject was achieved using an envelope method that was also stratified by gender to match as closely as possible the sequencing by gender. Each sequence was printed on a slip of paper and sealed into an individual envelope marked F or M. At the beginning of their first session, the subjects chose one of the available envelopes and this determined their unique sequence of interventions. Each subject completed eight intervention sessions spaced at least one week apart. 

Throughout the 21 minute intervention period, a curtain was positioned to prevent subjects from observing their right arm and the acupuncturist's activities. This both standardised and restricted interactions with the acupuncturist and facilitated his realistic application of simulated manipulation to an actual or virtual needle. The same acupuncturist applied all interventions; the 21 minute intervention period was standardised; manipulation was applied or simulated every three minutes; all subjects completed all study interventions and data were not analysed until the end of the study to avoid possible biases related to researcher expectations.

### 3.5. Intervention Procedure

Throughout each session, the subject lay supine on the treatment table. Prior to receiving each intervention, as part of the broader research program, a standardised series of baseline pressure pain threshold measurements were recorded from ten regional sites. The study's acupuncturist (with >35 years of clinical experience) then initiated the 21-minute intervention protocol for this arm of the research, summarised in the following timelines.


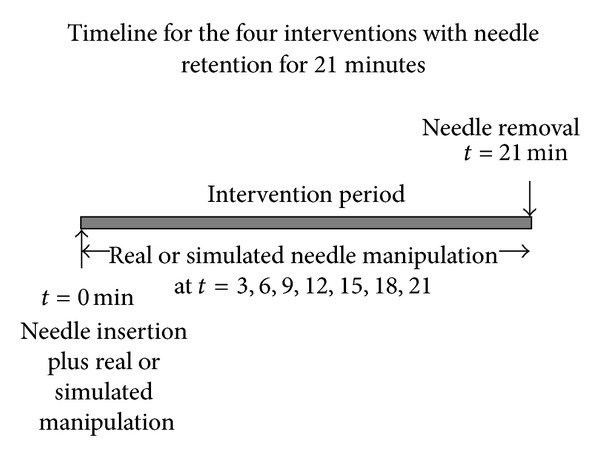

- Real or simulated needle “manipulation” at *t* = 0, 3,6, 9,12,15,18,21.
- VAS pain and needle sensation scores recorded at *t* = 1, 4,7, 10,13,16,19,22.

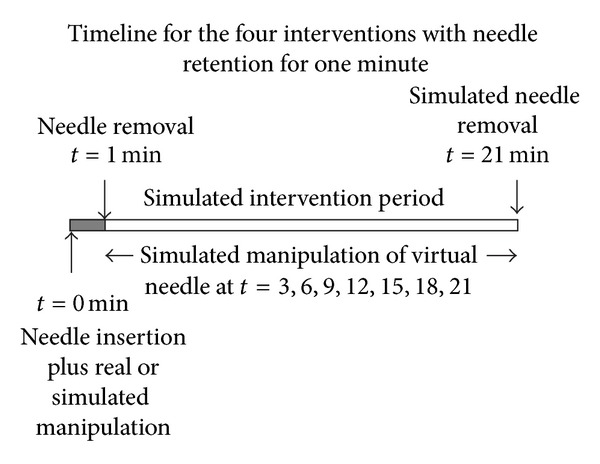

- Real or simulated needle “manipulation” at *t* = 0.
- Simulated “needle” manipulation at *t* = 3, 6,9, 12,15,18,21.
- VAS pain and needle sensation scores recorded at *t* = 1, 4,7, 10,13,16,19,22.

### 3.6. Statistical Analyses

Statistical analyses comprised one way ANOVA for correlated samples with Tukey post hoc testing, Chi square I (goodness of fit), and basic descriptive statistics for each time interval and intervention.

## 4. Results

### 4.1. Needle Sensation and Pain Intensity Profiles during the Intervention Period

**Figure 2:**
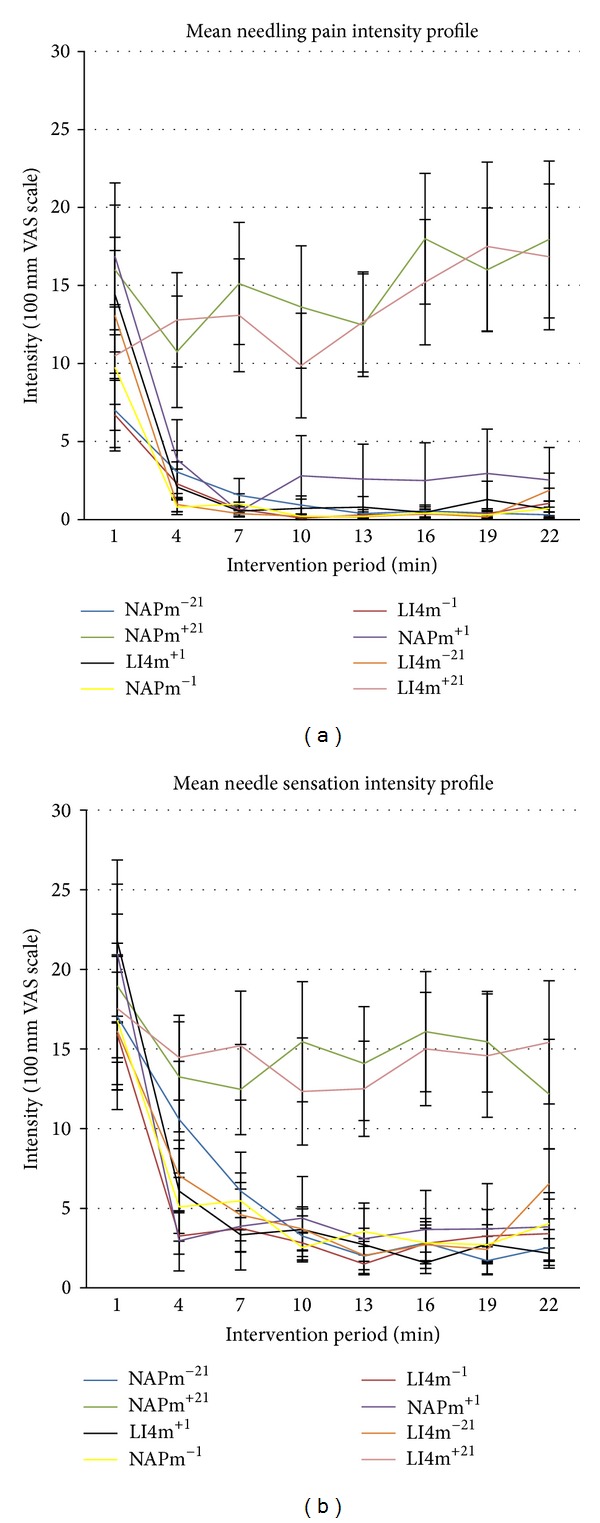
The mean pain intensity scores (left hand graph) and the mean sensation intensity scores (right hand graph) for the eight interventions at three-minute intervals during the 21-minute intervention period. The error bars depict ±1 standard error of the mean. The same colour key applies to both graphs.

In Figure 2, the left hand graph displays the mean needling pain intensity scores (% of 100 mm VAS) for the eight interventions at three-minute intervals during the 21-minute intervention period. The right hand graph presents the mean needle sensation intensity scores similarly. 

At time *t* = 1, there was no statistically significant difference between the interventions for either the mean VAS scores for pain (ANOVA *F*_7,161_ = 1.74, *P* = 0.103) or for needle sensation (ANOVA *F*_7,161_ = 0.48, *P* = 0.85). Mean values ranged from 6.7% to 16.9% for pain and 15.9% to 21.8% for needle sensation. However at the subsequent time intervals, these elevated levels were either maintained (for two interventions) or fell away rapidly for the remaining six. As a result, at time intervals *t* = 4 through to *t* = 22, there were multiple statistically significant differences among the means for pairs of interventions for both needle pain (ANOVA *F* statistic values between 5.33 and 12.54, *P* = 0.000 in all cases) and needle sensation means (ANOVA *F* statistic lay between 4.07 and 9.19, *P* = 0.000 in all cases). For both sets of profiles, post hoc Tukey testing revealed similar statistically significant patterns that included the following main features. Both interventions that involved 21-minute needle retention and ongoing manipulation maintained similarly elevated mean VAS scores that did not differ statistically significantly from each other, at each measurement period (in all cases for both pain and needle sensation, the values of the ANOVA *F* statistics lay between 1.48 and 0.0, with associated *P* values of between 0.24 and 0.95). For pain the mean elevations were between 16% and 18% and for needle sensation were between 12% and 19%.

With respect to both needling pain and needle sensation, the sets of profiles for the remaining six interventions showed similar, rapid decreases in mean scores. In all intervention comparisons, for pain and needle sensation, the values of the ANOVA *F* statistics for the individual measurement times lay between 1.94 and 0.27, with associated *P* values of between 0.09 and 0.93. By *t* = 4, the mean pain scores were <4% for all six intervention profiles and for needle sensation the mean scores were <7% by *t* = 7.

These two patterns within the sets of profiles reflected statistically significant differences. With the pain profiles, post hoc analyses (Tukey post hocs, in all cases statistical significance at least *P* < 0.05) showed that the LI4m^+21^ intervention mean VAS levels were statistically significantly higher than those for the six interventions that did not involve 21-minute duration and manipulation, for all time intervals from *t* = 4 to *t* = 22, with the single exception of NAPm^+1^ at *t* = 10. For the similar NAPm^+21^ intervention, the mean VAS scores were also statistically significantly higher than all other interventions from *t* = 7 to *t* = 22 and also for LI4m^−21^ and NAPm^−1^ at *t* = 4.

The needle sensation profiles for both LI4m^+21^ and NAPm^+21^ showed similar patterns to those for needling pain. However, there were more comparisons where their mean increases did not differ statistically significantly from the means for the remaining six interventions. This was the case for both interventions for four comparisons at *t* = 4 (LI4m^+1^, NAPm^−1^, LI4m^−21^, NAPm^−21^) and for one at *t* = 7 (with NAPm^+1^) and at *t* = 22 (with LI4m^−21^). There was one additional nonsignificant comparison involving LI4m^+21^at *t* = 10 (with NAPm^+1^) and seven involving NAPm^+21^, comprising three at *t* = 7 (NAPm^−21^, LI4m^−21^, NAPm^−1^) and four at *t* = 22 (LI4m^−1^, NAPm^+1^, LI4m^+1^, NAPm^−1^).

In summary, the continued application of needle manipulation and retention of the needle were important for maintaining elevated needle sensation as well as pain associated with needling. By contrast, both the needle pain and sensation experienced was independent of insertion site.

### 4.2. Relation between Pain and Needle Sensation Perceptions

The following four graphs show for each intervention, the number of subjects at each three-minute recording interval who reported experiencing each of the following: neither pain nor needle sensation; both pain and needle sensation; only pain; or only needle sensation.

**Figure 3:**
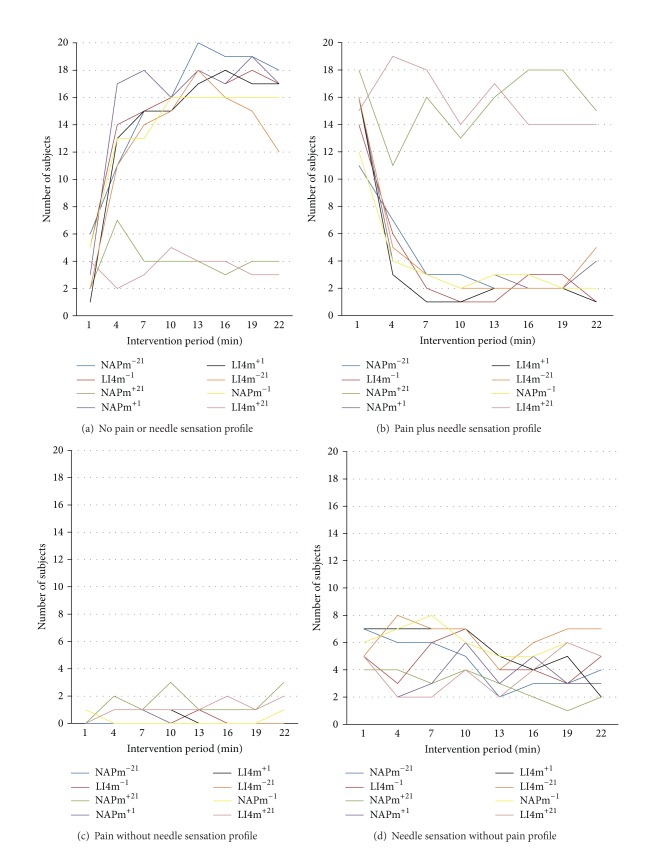
Comparison of the eight interventions with respect to the number of subjects at each three-minute recording interval who had: neither pain nor needle sensation (a); both pain and needle sensation (b); only pain (c); or only needle sensation (d). In all cases, total number of subjects = 24.

From the frequencies of subjects among interventions reporting neither pain nor needle sensation (Figure 3(a)) or both pain and needle sensation (Figure 3(b)), the profiles for the pair of interventions that included needle retention and ongoing manipulation during the 21-minute period are clearly different from the remaining six interventions, for the time intervals other than *t* = 1 following the initial needle insertion. For this pair, most subjects experienced both pain and needle sensation at each interval; while for the remaining six, the reverse was the case, with most subjects not experiencing either pain or needle sensation. These frequencies were similar across the 21-minute intervention period. The profiles for simultaneous presence of pain and needle sensation (Figure 3(b)) are strikingly similar to the profiles shown in Figure 2 for both the mean needle sensation intensity and the mean pain intensity.

By contrast, virtually no subjects reported pain alone (Figure 3(c)) for any intervention or time interval. Similarly, needle sensation in the absence of pain (Figure 3(d)) was only experienced by a small proportion of subjects at any time interval. It is interesting that although four of the interventions involved only one minute of needling there were still several reports of pain and/or needle sensation throughout the entire 21 minute reporting period.

In summary, the experiences of needle sensation and pain were closely linked with respect to duration and presence of manipulation but not to location of needling.

**Figure 4:**
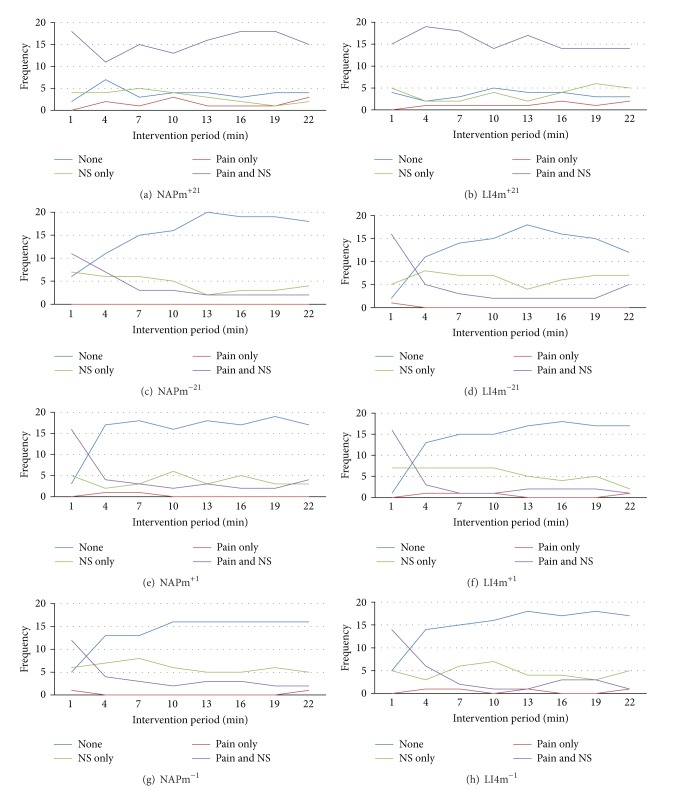
Comparison within each of the eight interventions of the number of subjects at each three-minute recording interval who had neither pain nor needle sensation; both pain and needle sensation; only pain; or only needle sensation. Total number of subjects = 24.

From the pain and needle sensation profiles for individual interventions shown in Figure 4, all interventions had two common features. At *t* = 1 (when all had the common experience of a needle being inserted and retained for one minute) significantly more subjects reported the presence of both pain and needle sensation than other possibilities (*P* < 0.05 for all eight interventions, Chi square I). The reporting of pain alone was either absent at most of (LI4m^−21^, NAPm^+1^, NAPm^−1^) or even all of the three-minute measurement periods (NAPm^−21^).

The eight profiles clustered into two distinct two response patterns: one shared by the pair with 21-minute needle retention and repeated manipulation, and the other by the remaining six interventions.

Pattern 1: for all measurement intervals including *t* = 1, significantly more subjects reported the presence of both pain and needle sensation (*P* < 0.05, Chi square I).

Pattern 2: for all measurement intervals except *t* = 1, significantly more subjects reported absence of both pain and needle sensation (*P* < 0.05, Chi square I). This applied whether the needles were retained for one minute or 21 minutes; and with the one-minute retentions, whether or not manipulation was applied. Therefore, again the distinguishing parameter values were needle retention and application of manipulation but not site of insertion.

### 4.3. Qualities of Needle Sensation: Needling Sensation Descriptors

At the end of each session, subjects reported the needling sensations they had experienced during the intervention. Note that subjects were not limited to a single descriptor. Since these unsolicited descriptors reported by subjects were found to be in good agreement with ones from the MMPQ, they have been grouped according to MMPQ categories [4, 5]. This system addresses both quality and intensity of a descriptor, so that interventions could be compared in terms of both the number and the intensity of descriptors used, both within individual MMPQ descriptor categories and overall. Three additional categories were created for unrepresented terms: “warm” (since the MMPQ category commences with “hot”), “electricity”, “and can't describe.”

The categories that contain descriptors provided by subjects are shown in Table 2.

**Figure 5:**
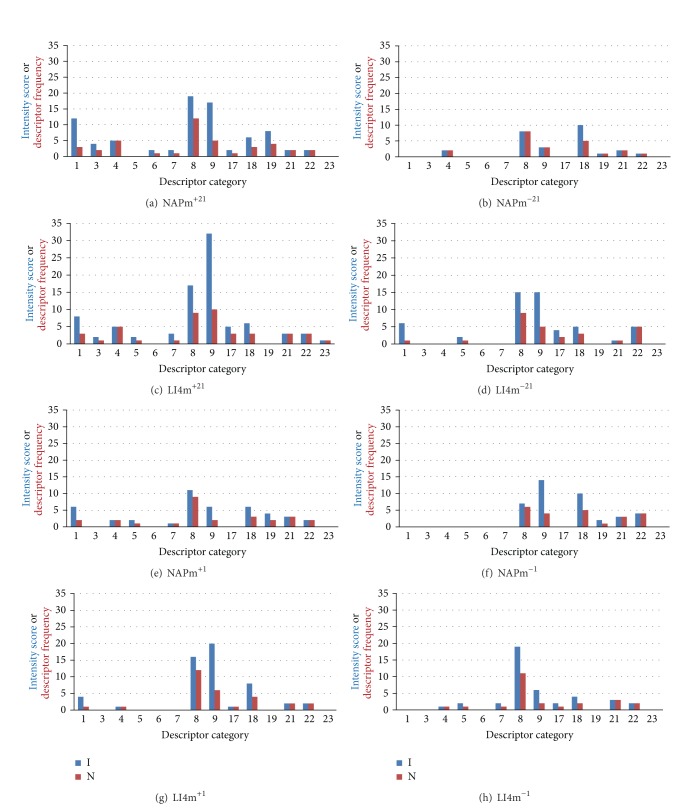
Profiles for each intervention for the frequency (*N*) and intensity (*I*) of sensory descriptors reported from each descriptor category list. (1) flickering, pulsing, quivering, throbbing, beating, pounding; (3) pricking, boring, drilling, stabbing; (4) sharp, cutting, lacerating; (5) pinching, pressing, gnawing, cramping, crushing; (6) tugging, pulling, wrenching; (7) hot, burning, scalding, searing; (8) tingling, itchy, smarting, stinging; (9) dull, sore, hurting, aching, heavy; (17) spreading, radiating, penetrating, piercing; (18) tight, numb, squeezing, drawing, tearing; (19) cool, cold, freezing; (21) electricity; (22) warm; (23) indescribable.

Study subjects' results are summarised in Table 3 and Figure 5. Among the eight interventions, more descriptors (41 and 43) were reported for the pair of 21-minute interventions with manipulation compared with the other six interventions (22 to 29). The different numbers of descriptors reported are not explained by differing numbers of reports of no sensation among the eight interventions. For the pair of one-minute interventions without manipulation, the number and intensity scores are identical and are also similar to those for the one-minute NAP intervention with manipulation (NAPm^+1^). The intensity and number of descriptors for the one-minute LI4 with manipulation (LI4m^+1^) closely resembles the findings for the 21-minute LI4 without manipulation intervention (LI4m^−21^).

The MMPQ based descriptor intensity profiles are shown for the eight interventions in Figures 5(a)–5(h). The MMPQ categories relevant to these results are listed in the caption together with the three ungrouped additions of electricity, warm, and indescribable.

For all eight interventions, descriptors were reported from the same five descriptor categories that included 8, 9, 18, and the additional 21 (electricity) and 22 (warm). The most frequently reported descriptors were from category 8 and included some form or intensity of tingle, sting or itch. The second most frequently used terms were from category 18 (typically numbness). Less frequent but reported for all interventions were category 9 terms (dull ache). Far less frequent were the ungrouped terms “warm” and “electricity.” 

Figure 5 indicates that, in general, the descriptor profiles were very similar for each pair of equivalent LI4 and NAP interventions. The only minor exception was that category 9 terms were reported more frequently among the LI4 interventions. The two LI4 interventions involving manipulation had the highest intensity scores among all interventions for category 9 terms and for the four LI4 interventions, category 9 terms were reported 21 times compared with 12 times for the four NAP interventions. However these reports only involved a minority of subjects for the four interventions at either site (20 reports compared with 12).

## 5. Discussion

The findings for both needle sensation and pain among the eight interventions are strikingly consistent in terms of providing both positive and negative instances, all of which support the conclusion that needle manipulation and needle retention are important for maintaining an elevation in needle sensation and pain. By contrast, no additional or differential effect was shown for the site of needling insertion although one was an acupoint (LI4) and the other was not (NAP). These findings related to both quantitative VAS scores as well as to the qualitative descriptors spontaneously reported by subjects and discussed later in this section. 

Another clear relationship among the findings was that needling pain and needle sensation overwhelmingly were present or absent together. This may relate to the role of acute pain in helping to protect the body. Its role is one of warning and alerting the conscious organism about the presence of a noxious or potentially harmful sensory stimulus. This is demonstrated by the similar role of pain across such diverse perceptual experiences as, for example, touch, sound, light, or taste. Therefore, piercing the intact skin and underlying tissues with a needle represents an invasive threat and should activate appropriate sensory mechanisms. Deep piercing together with needle manipulation, by involving more stimulation would augment the sensory input and be expected to produce a more intense sensory perception of discomfort and pain. Since “*deqi*” or needle sensation is encouraged by mechanical manipulation and deep needle insertion (as opposed to shallow insertion without manipulation), it would be expected that pain would also be elicited. Therefore, pain or discomfort would be a likely accompaniment to “*deqi.*” Is it possible, for example, that the original concept of *deqi* embraced the whole range of sensations elicited by needling, including acute pain? What is not known from the early literature is whether originally *deqi* was ever demonstrated—*as opposed to being assumed*—to be restricted solely to acupoints, rather than an experience associated with needling living tissues more generally. 

The qualitative descriptors used by the subjects in this study were the subjects' own individually and spontaneously provided words. Therefore it is noteworthy first, that the profiles for qualities of needle sensation were similar for NAP and LI4 and second, that the terms fitted almost perfectly into the category groupings developed for the well validated MMPQ [4, 5]. Moreover, both the number of descriptors as well as the intensities of the descriptors used for the two interventions that produced the significantly higher needle sensation and pain scores during the interventions (LI4m^+21^ and NAPm^+21^) were higher than for the remaining six interventions. Again, while these two findings did not differentiate between acupoint and nonacupoint locations, they did provide another linking of pain with needle sensation. 

MacPherson and Asghar [9] developed a qualitative and quantitative classification of needle sensations associated with *deqi* based on ratings by 20 TCM acupuncture experts. Two clusters of sensations were identified. One cluster was linked with *deqi* and comprised seven sensations: aching, dull, heavy, numb, radiating, spreading, and tingling. The second cluster related to acute needling pain and included nine sensations: burning, hot, hurting, pinching, pricking, sharp, shocking, stinging, and tender. In the present study, it is noteworthy that the needle sensation descriptors primarily fell into these authors' *deqi* descriptor cluster. All eight interventions reported descriptors from five descriptor categories, comprising 8, 9, 18 and the ungrouped additional “warm” and “electric.” The most frequently reported descriptors among interventions were from category 8 and included some form or intensity of tingle, sting, or itch. The second most frequent terms were from category 18 (typically numbness). Less frequent but reported for all interventions were category 9 terms (dull ache). Far less frequent were the ungrouped terms “warm” and “electricity.” These findings do support the notion that subjects were, at least primarily, discriminating and reporting on needle sensation, rather than pain. 

The study also showed that needle sensation was maintained only when the needle was both retained and received ongoing manipulation (Figure 2). A plausible explanation is that mechanical manipulation causes injury to the tissues around the needle and one of the body's reactions to this injury is pain (needle sensation or *deqi*). This may contribute to activation of the body's defensive system by increasing blood flow to the site of insertion [20], which in turn modifies delivery of oxygen, neurohumoral and anti-inflammatory mediators to the site [21]. 

Subjects spontaneously provided needle sensation descriptors that also describe pain: qualitatively and quantitatively and relevant here is the concept of “pain threshold,” that is, the intensity of a nonpainful sensory stimulus when it begins to take on *the beginnings of discomfort, the beginnings of pain. *Obviously the stimulus quality prior to this level was not perceived as painful. An individual's pain threshold is not constant and experimental studies have confirmed the enhancing effect of anxiety on ratings of pain intensity [22], unpleasantness [23] and pain threshold [24]. In response to experimental cold pressor pain stimulation, McCaul and Haugtvedt [25] found that distraction is a better coping strategy than attention to sensations when subjects are asked to report pain threshold and tolerance. Wagner and colleagues [26] reported that induced sad effect leads to reduced heat pain thresholds in healthy subjects. This was regarded as probably due to altered lateral thalamic activity, which is potentially associated with changed attentional processes. 

The descriptors in the MMPQ are not the sole preserve of pain. They are merely descriptors of sensory experiences, in terms of quality and intensity, and may not necessarily be describing something that is unpleasant or potentially noxious. Even some of the more intense descriptors may, in some sensory experiences and in certain settings, reflect positive and very pleasurable sensations in healthy individuals, as for example with the pressure of deep, strong massage or the spreading and radiating heat from a heat lamp. 

Pain may contribute to “*deqi*” with respect to clinical effects associated with needling, given the linking of pain with endogenous endorphin system activation. Certainly it has been extensively demonstrated that pain induces the synthesis of beta endorphins by the pituitary gland and when released, these affect the central and peripheral nervous system and relieve pain by binding to specific opioid receptors in these areas [27].

Vincent and colleagues' early study [6], using a modified MMPQ, found that similar levels of needle sensation were produced at both acupoints and nonacupoints, suggesting that *deqi* was not exclusive to acupoints. The present study strongly supports these findings. For both acupoint and nonacupoint with manipulation and a needle retention time of 21 minutes (LI4m^+21^ and NAPm^+21^), the level of needle sensation remained constant at around 15% compared to the other interventions which dropped below 5% (Figure 2). These findings were evident both quantitatively, from VAS sensation intensity scores during the needling period, and qualitatively, from the sensation descriptors provided by subjects (Figures 5(a) and 5(c) and Table 3).

Typically and necessarily, studies of needle sensation have involved healthy study subjects. This is perhaps incongruous, given that the intent of clinical acupuncture interventions is to restore balance or health when there is some imbalance or illness. That is, is it appropriate to assume that needle sensation may be linked in either a causative or a correlative manner with a specific, measurable physiological response; and if so, what clinical response(s) could be regarded as being appropriate to examine in relation to presence or levels of needle sensation in a healthy subject? Pain threshold has been a common choice here. Not only can it be quantified with VAS and MMPQ style instruments, but measurement is neither invasive nor injurious to tissues. However, it may be regarded as counter intuitive that acupuncture, a process hypothesised to restore bodily functional balance, should modify the resting pain threshold in a healthy individual. On the other hand, if needle sensation is regarded as simply part of the sensory system's alerting of the presence of an invasive, potentially noxious insult to the tissues, then the recruitment of defences would be typical and expected. 

It is important to stress that the subjects in this study were selected on the basis of being in good health, since the aim of the study was to obtain baseline information about the influence of the three parameters being researched. Whether the responses to the same set of interventions would be different for patients with specific clinical conditions is unknown. However, the profiles and other data collected in the present research could serve as a baseline for related clinically oriented research. 

In the present study, there was little evidence of a significant placebo effect in that in general, subjects did not report further pain or needle sensation after needle removal in the one minute retention interventions. The limited number and range of sensations reported after needling for these interventions typically included numbness and tickling/tingling and Figure 4 shows that needling sensation and pain were reported by a small minority of these subjects throughout the 21 minute intervention period. It is possible that numbness or tingling experienced after needling may be due to the arm and hand being left immobile for 21 minutes and rather than being a placebo effect, it is an actual physiological response to this unnatural inactivity. Our group had previously encountered a similar phenomenon in a study where subjects received, as the control intervention, inactive laser, with some subjects reporting feelings of heaviness, numbness, and tickling/itching [19]. These findings suggest that studies may need to take such factors into account, not only when considering “placebo” responses, but also with respect to subjects' perceived responses to potentially “real” interventions.

## 6. Conclusion

This study examined three needling parameters (site of insertion, manipulation, and retention time) in relation to the outcome measures of intensity of pain or needle sensation and qualitative descriptors of the needle sensation. Results showed that while the levels of needle sensation and pain were similarly intense following needle insertion for all interventions, initial intensity levels faded away rapidly unless the needle was both retained and manipulation repeated. Neither the eliciting nor maintaining of needle sensation or pain was restricted to a designated acupoint, with similar outcomes obtained at both LI4 and NAP. Typically, both pain and needle sensation were present (or absent) together and very few subjects reported pain or needle sensation in isolation.

The needle sensation descriptors spontaneously reported by subjects were in good agreement with the MMPQ pain descriptors. Based on the MMPQ categories, the descriptors reported by subjects did not differentiate between the two needling sites in terms of either quality or the intensity of the terms used. However, they did discriminate between the two 21-minute interventions with manipulation present, compared with the other six interventions. More descriptors and greater intensity scores were reported for the former pair of intervention compared with other interventions, all of which reported very similar lower intensity scores and numbers of descriptors.

## Figures and Tables

**Table 1:** 

Intervention	Site	Retention time	Manipulation
LI4m^+1^	LI4	1 minute	Present
LI4m^−1^	LI4	1 minute	Absent (simulated manipulation)
LI4m^+21^	LI4	21 minutes	Present
LI4m^−21^	LI4	21 minutes	Absent (simulated manipulation)
NAPm^+1^	NAP	1 minute	Present
NAPm^−1^	NAP	1 minute	Absent (simulated manipulation)
NAPm^+21^	NAP	21 minutes	Present
NAPm^−21^	NAP	21 minutes	Absent (simulated manipulation)

**Table 2:** 

Category	Descriptors (in order of intensity rank)
1	Flickering, pulsing, quivering, throbbing, beating, pounding
3	Pricking, boring, drilling, stabbing
4	Sharp, cutting, lacerating
5	Pinching, pressing, gnawing, cramping, crushing
6	Tugging, pulling, wrenching
7	Hot, burning, scalding, searing
8	Tingling, itchy, smarting, stinging
9	Dull, sore, hurting, aching, heavy
17	Spreading, radiating, penetrating, piercing
18	Tight, numb, squeezing, drawing, tearing
19	Cool, cold, freezing
21	Electricity
22	Warm
23	Indescribable

**Table 3:** Total Number (*N*) and intensity (*I*) of sensory descriptors reported for the eight interventions (no sensation responses are shown in parentheses).

Intervention	NAPm^+^		NAPm^−^		LI4m^+^		LI4m^−^	
	*I*	*N*	*I*	*N*	*I*	*N*	*I*	*N*
21 min	81	41(2)	33	22(7)	87	43(2)	53	27(6)
1 min	43	27(3)	41	24(7)	55	29(6)	41	24(7)
